# Management of rifampicin mono‐resistant tuberculosis in Queensland, Australia: a retrospective case series

**DOI:** 10.1002/rcr2.366

**Published:** 2018-09-14

**Authors:** Justine Gibson, Ellen Donnan, Geoffrey Eather

**Affiliations:** ^1^ Department of Respiratory and Sleep Medicine Princess Alexandra Hospital Queensland Australia; ^2^ Metro South Clinical Tuberculosis Service Princess Alexandra Hospital Queensland Australia; ^3^ Communicable Diseases Branch Queensland Department of Health Queensland Australia

**Keywords:** Drug‐resistant tuberculosis, mycobacterium tuberculosis, rifampicin mono‐resistance

## Abstract

Rifampicin mono‐resistant tuberculosis (RMR‐TB) is rare worldwide; however, it is associated with poor treatment outcomes. Evidence to guide the treatment of RMR‐TB is lacking. International guidelines have recently changed and now recommend that RMR‐TB should be managed with multi‐drug‐resistant tuberculosis (MDR‐TB) regimens. This report is a retrospective review of all cases of RMR‐TB in Queensland, Australia, from 2000 to 2016 to assess treatment outcomes and regimens used. Twelve cases of RMR‐TB were diagnosed, with seven patients completing treatment in Queensland. This study confirms that RMR‐TB is rare in Queensland. Generally extended regimens with first‐line agents +/− a fluoroquinolone were used, and all patients who completed treatment in Queensland had successful outcomes.

## Introduction

Drug‐resistant tuberculosis (TB) is an emerging problem, making TB management more challenging. Rifampicin is a key agent in TB regimens. Resistance to this agent is most commonly associated with resistance to isoniazid, designated multi‐drug‐resistant tuberculosis (MDR‐TB). Rifampicin resistance without resistance to the other first‐line TB agents is referred to as rifampicin mono‐resistant tuberculosis (RMR‐TB). RMR‐TB is rare in Australia and internationally 1, 2, 3. In 2014, the World Health Organization (WHO) estimated that 1.1% of patients with TB worldwide had rifampicin resistance without additional resistance to isoniazid 3. In Australia, RMR‐TB accounted for 0.3% of Australian TB notifications in 2013 compared to MDR‐TB at 2.4% 1.

RMR‐TB has been associated with a high risk of treatment failure, with a previous observational study suggesting that only two‐thirds of patients had successful treatment outcomes 4. Although it is widely accepted that regimens without rifampicin are associated with poor outcomes, evidence to guide the management of RMR‐TB is lacking. A recent systematic review highlighted the lack of data in the treatment of RMR‐TB, and no conclusion regarding the efficacy of treatment regimens could be made 5.

Despite the lack of evidence, guidelines in the management of RMR‐TB have changed over the last decade. In 2003, the American Thoracic Society, Centers for Disease Control and Infectious Diseases Society of America recommended a 12–18‐month regimen for RMR‐TB with isoniazid, ethambutol, and a fluoroquinolone, with the addition of pyrazinamide for the first 2 months 6. The most recent WHO update in 2016 recommends that rifampicin‐resistant TB, regardless of resistance to isoniazid (or other first‐line agents), should be treated as per MDR‐TB guidelines 7. By this definition, RMR‐TB should be included in this recommendation. This requires a 9–12‐month short course regimen or a longer conventional MDR‐TB regimen of 18–20 months in some cases. Both regimens use multiple second‐line anti‐TB agents, including an injectable agent for 4–8 months, and are associated with increased toxicity and cost 8, 9.

The treatment regimens in Queensland, Australia, for RMR‐TB are not standardized. Our study was performed to analyse all cases of RMR‐TB in this state over the defined period to describe the frequency of this condition and the treatment regimens utilized with reference to outcomes and adherence to accepted guidelines.

## Case Series

### Methods

This retrospective case series identified all cases of rifampicin‐resistant TB without isoniazid resistance in Queensland from 1 January 2000 to 31 December 2016. These cases were identified and managed in multiple sites throughout Queensland by different attending clinicians.

RMR‐TB is defined as a resistance to rifampicin without resistance to any other first‐line TB agents. In this case series, we included patients with streptomycin resistance but none who demonstrated resistance to isoniazid, pyrazinamide, or ethambutol. We acknowledge that the patients with streptomycin resistance do not meet the definition of RMR‐TB; however, given that streptomycin is rarely used in clinical practice, we have included these patients for analysis.

Data were obtained from the Queensland Notifiable Conditions System (NoCS) (extracted 6 December 2017), a government database for the surveillance of communicable diseases. All included cases had confirmatory testing and drug susceptibility testing (DST) performed at the Queensland Mycobacterial Reference Laboratory.

Baseline characteristics, prior TB treatment, HIV status, sputum smear results, radiology, DST profile, treatment regimens, duration, and outcomes were obtained from NoCS and cross referenced with medical records. Treatment outcomes were assessed in accordance with the WHO 2014 updated definitions 10.

### Results

Twelve confirmed cases of RMR‐TB were identified. The mean age was 29.1 ± 12.0 years, with seven females. All patients were overseas born, with five patients from Papua New Guinea. Nine cases (75%) had pulmonary disease, and four of these had cavitary disease. Six (66.7%) were smear positive on direct sputum microscopy. One patient had a history of prior treatment for TB. Eleven cases (92%) were HIV negative, and one patient’s HIV status was unknown.

All patients had DST, which confirmed that all isolates were resistant to rifampicin and sensitive to other first‐line oral agents (isoniazid, pyrazinamide, and ethambutol). Two cases (16.7%) had resistance to streptomycin. Only four cases had DST to second‐line agents performed. One case was resistant to ofloxacin and one resistant to ethionamide, but otherwise, these four isolates were sensitive to the standard second‐line TB agents that are tested in Queensland (ofloxacin, amikacin, ethionamide, para‐aminosalicyclic acid, capreomycin, kanamycin, and cycloserine).

Complete data on treatment and outcomes were available for seven of the 12 cases (Table 1). Of the patients not included in Table 1, one patient died of TB prior to commencing treatment, and four patients were transferred out and did not complete treatment in Australia. Final outcome data for these four patients were not available.

**Table 1:** Treatment regimens and outcomes for all patients with rifampicin mono‐resistant TB who underwent treatment in Queensland, Australia.

Age (years)/gender	HIV status	Site of disease	Smear status	Cavitary disease	Treatment Regimen	Total duration (months)	Outcome*
35/male	Negative	Extrapulmonary (lymph node)	Negative	—	2 HRZE, 13 HE	15	Treatment completed
33/female	Negative	Extrapulmonary (lymph node)	Negative	—	2 HRE, 10 HZE	12	Treatment completed
34/female	Negative	Extrapulmonary (gastrointestinal)	Negative	—	19 HZE	19	Treatment completed
33/male	Negative	Pulmonary	Negative	Yes	1 HRZE, 3Mfx HZE, 1Mfx HE, 13 Mfx H	18	Treatment completed
26/male	Negative	Pulmonary	Negative	Yes	2 HRZE, 12 Mfx HZE	14	Treatment completed
32/male	Negative	Pulmonary	Positive	Yes	1 Mfx HZE, 11 Mfx HE	12	Treatment success
20/female	Negative	Pulmonary	Positive	Yes	2 Amk Mfx HZE, 5 Mfx HZE, 5 HZE	12	Treatment success

Amk, amikacin; E, ethambutol; H, isoniazid; Mfx, moxifloxacin; R, rifampicin; Z, pyrazinamide.

* Outcomes defined by WHO for rifampicin‐resistant TB 10.

To date, all the patients who completed treatment in Queensland had successful outcomes. Six patients who completed treatment have received over three years of surveillance without relapse; one patient has completed two years post‐treatment surveillance.

Isoniazid and ethambutol were given for the complete treatment course in six of seven (86%) cases, and the majority of patients (86%) received a minimum of two months of pyrazinamide. Overall, the majority of patients were treated with the first‐line agents (isoniazid, ethambutol, pyrazinamide), and moxifloxacin was added to the regimen in four cases. Only one patient received intravenous amikacin, and this was for a duration of two months. The mean duration of treatment was 14.6 months (range 12–19 months).

## Discussion

While this study is small in size and has limitations, it does confirm that RMR‐TB is rare in Queensland, and although antimicrobial regimens have varied, treatment outcomes have been successful. Generally, a minimum 12‐month treatment regimen with the first‐line agents +/− a fluoroquinolone was used. Isoniazid and ethambutol were the backbone of treatment regimens, and 86% of patients received a minimum of two months of pyrazinamide. One patient ceased pyrazinamide after one month due to drug induced hepatotoxicity. All three cases of extrapulmonary TB were managed without a fluoroquinolone, which was a variation from international guidelines at the time. Despite this, all extrapulmonary RMR‐TB cases achieved positive outcomes. MDR‐TB regimens have not been historically used in Queensland, as expected, given that this is a relatively new recommendation.

Regimens consisting of first‐line agents +/− fluoroquinolones appear to achieve good outcomes, with 100% success rates in patients who completed treatment in Queensland. Similar results were found in a South Korean study, which showed that a regimen with first‐line drugs and a fluoroquinolone had similar outcomes to MDR‐TB regimens, with no recurrence recorded in either group 11.

The median duration of treatment in our study was 14.6 months. In addition, only one patient in this cohort received an injectable agent, and this was for two months. While our data suggest good outcomes without the use of injectable agents (in all but one case) or other second‐line agents (apart from fluoroquinolones), the overall duration is longer than the short‐course MDR‐TB regimen. The short‐course MDR‐TB regimen, however, still requires four to six months of an injectable agent as well as three second‐line oral agents, potentially increasing cost and toxicity, including potential ototoxicity associated with prolonged treatment with aminoglycosides 9. In well‐resourced settings such as Queensland, where access to high‐quality DST is available, the recommendation for MDR‐TB regimens for RMR‐TB cases may not be necessary, a suggestion that is supported by our data.

This case series does not include any HIV‐infected patients. HIV‐positive patients are more likely to present with extrapulmonary and disseminated disease, and although there are patients with extrapulmonary disease in this cohort, our results may not be applicable to HIV‐infected patients.

Rifampicin resistance is frequently regarded as a proxy for MDR‐TB as the majority of rifampicin‐resistant strains will have isoniazid resistance. One of the arguments to support using a MDR‐TB regimen for all rifampicin‐resistant TB is the rapid identification of TB isolates using the GeneXpert to detect rpoB gene mutations. Rapid diagnostic tests for isoniazid resistance and full DST are not as reliable or accessible in all countries, resulting in the recommendation to use a MDR‐TB regimen as the preferred treatment in the presence of rifampicin resistance. Although the prevalence of RMR‐TB is rare worldwide, there is geographical variation, and RMR‐TB can account for up to 10% of TB infections in certain areas 12. As there are efficacious and potentially less‐toxic regimens for RMR‐TB when compared to MDR‐TB regimens, it seems important to differentiate the two resistance profiles in settings that have the resources to do so reliably.

Management of RMR‐TB is an area where further studies are required. Our study is too small to provide evidence of the optimal treatment regimen for RMR‐TB but does suggest that good outcomes can be obtained with regimens constructed around first‐line agents and the addition of a fluoroquinolone. Ideally, randomized controlled trials are needed to determine the efficacy of treatment regimens and the optimal duration of therapy. However, given the low incidence of RMR‐TB worldwide, this will be challenging. Although our study is small, it suggests that the literal interpretation of the WHO guideline may not be applicable in many cases where diagnostic resources are available to confirm true RMR‐TB. Given the increasing incidence of drug‐resistant TB worldwide, further studies in this area should be a priority.

### Disclosure statement

Ethics approval for this study was obtained from the Metro South Human Research Ethics Committee (HREC/17/QPAH/175).
